# A Comparative Analysis of Bacterial Communities in Settled Air Dust and Vacuumed Surface Dust From University Dormitories and Associations With Respiratory Health

**DOI:** 10.7759/cureus.99514

**Published:** 2025-12-18

**Authors:** Xi Fu, Xinghan Wang, Dan Norbäck, Xin Zhang, Yu Sun

**Affiliations:** 1 School of Public Health, Guangdong Pharmaceutical University, Guangzhou, CHN; 2 College of Life Sciences, South China Agricultural University, Guangzhou, CHN; 3 Department of Medical Science, Uppsala University, Uppsala, SWE; 4 Institute of Environmental Science, Shanxi University, Taiyuan, CHN

**Keywords:** airborne dust, asthma, bacterial diversity, indoor microbiome, sampling methods

## Abstract

Background: The indoor microbiome significantly impacts human health. Different sampling methods are used to characterize this environment, but it is unclear how these methods affect the resulting microbial profiles and health-related interpretations. This study aimed to compare the bacterial communities captured by two common methods, i.e., passive collection of settled air dust and active vacuuming of surface dust, and to evaluate how sampling choice influences epidemiological associations with respiratory health.

Methods: We collected paired settled air dust (n = 86) and vacuumed surface dust (n = 83) samples from 87 university dormitory rooms. The bacterial composition was characterized by sequencing the V3-V4 region of the 16S rRNA gene. We analyzed differences in bacterial diversity, taxonomic composition, predicted functional profiles, and associations with self-reported student health data (rhinitis, asthma, and respiratory infections).

Results: The two sampling methods captured drastically different bacterial communities (PERMANOVA R² = 0.65, p < 0.001). Vacuumed dust samples were dominated by the genus *Pseudomonas* (75.1% mean relative abundance), whereas air dust samples were more diverse and primarily composed of *Ralstonia* (15.6%), *Pelomonas* (11.3%), and *Anoxybacillus* (9.3%). Air dust samples exhibited significantly higher alpha diversity (observed taxa: 906.2 ± 179.6) compared to vacuumed dust (345.1 ± 82.3, p < 0.001). These compositional differences led to distinct predicted functional pathways and divergent associations with health outcomes. For asthma, airborne *Collinsella* was associated with strongly increased odds (OR 2.17, p = 0.003), while *Paracoccus* was associated with decreased odds (OR 0.062, p = 0.006). By contrast, associations in vacuumed dust were limited to taxa with small effect sizes, such as *Peptoclostridium* (OR 1.07, p = 0.004). Furthermore, while airborne genera like *Slackia* were associated with respiratory infections (OR 5.94, p = 0.005), no significant bacterial associations for respiratory infections were found in vacuumed dust.

Conclusion: The choice between sampling settled air dust versus vacuumed surface dust provides profoundly different views of the indoor bacteriome, which can lead to contradictory conclusions in epidemiological studies of asthma and respiratory illness. Our findings underscore that the selection of an environmental sampling strategy is a critical methodological decision that can dictate the outcome and potential health implications of indoor microbiome research. Researchers must align their sampling method with a clear exposure hypothesis to ensure valid health risk assessments. Future indoor air quality standards and epidemiological studies should consider implementing multi-method sampling to capture a comprehensive profile of microbial exposure.

## Introduction

The microbial communities of indoor environments, often referred to as the indoor microbiome, play a critical role in human health [1-3]. Given that people spend approximately 90% of their time indoors, exposure to these microbes can influence conditions such as asthma, allergies, and respiratory infections [4-10]. Culture-independent methods, particularly next-generation sequencing of the 16S rRNA gene, have revolutionized our ability to study these complex bacterial ecosystems [11].

A variety of sampling strategies are employed in indoor microbiome research, each with its own advantages and limitations. These methods broadly include surface sampling and airborne dust collection [12]. Surface sampling, such as swabbing or using vacuum cleaners with filters on floors and mattresses, is often used as it represents a long-term, integrated sample of microbial exposure and provides ample material for analysis. By contrast, airborne dust, which is thought to more closely represent inhalable exposure, can be collected passively via settled dust collectors like petri dishes and electrostatic dustfall collectors (EDCs), or actively with an air pump or BioSampler [12,13].

Despite the widespread use of these methods, direct comparisons using modern sequencing techniques are limited. Previous work has shown that different sampling approaches can yield varying estimates of fungal or bacterial composition[12,14]. This discrepancy is critical because the choice of method could fundamentally alter the interpretation of a study's results, particularly in epidemiological investigations seeking to link specific microbial exposures to health outcomes. For example, a microbe found in floor dust may not be easily aerosolized and inhaled, making its association with a respiratory condition questionable.

Therefore, this study was designed to directly compare the bacterial communities in dust collected by two common methods within the same indoor environments: passive collection of settled air dust and vacuuming of dust from floors and other surfaces. We hypothesized that these two methods would capture distinct bacterial communities, differing in diversity, taxonomic composition, and, consequently, in their associations with occupant health. Our specific objectives were (1) to determine if settled air dust and vacuumed surface dust yield distinct bacterial profiles and (2) to investigate whether these methodological differences lead to divergent conclusions regarding health associations with asthma and respiratory infections.

## Materials and methods

Study design and sample collection

Dust samples were collected from 87 rooms across 10 dormitory buildings at Shanxi University, Taiyuan, China, between November and December 2013. From each building, eight to 10 rooms were randomly selected. In each room, two types of dust samples were collected concurrently. For settled air dust collection, two sterile, open petri dishes were placed on a flat surface approximately 1.2 meters above the floor and left for seven days to passively collect settling dust. Vacuumed surface dust was collected on the first day of air dust collection. The surface dust was collected using a 400 W vacuum cleaner with a dust sampling device (ALK Abello, Copenhagen, Denmark) equipped with a 6 µm pore size Millipore filter. This filter retains approximately 74% of particles 0.3-0.5 μm, 81% of particles 0.5-1.0 μm, and over 95% of particles >1 μm [15]. A standardized four-minute vacuuming protocol was followed, which included two minutes on the floor and two minutes on other surfaces such as chairs, desks, and textile curtains. This procedure was conducted twice in each room (once near the window and once near the corridor), and the two filter samples were merged for subsequent analysis. Following collection, dust from the air dust petri dishes was suspended by washing with 2 ml of phosphate-buffered saline with Tween-20 (PBST). The vacuumed dust samples were processed in a biosafety cabinet by sieving through a sterile 0.3-mm mesh screen and then suspending the fine dust in PBST. Supernatants from both sample types were stored at -80°C until DNA extraction. In total, 86 air dust samples and 83 vacuumed dust samples yielded sufficient DNA for successful amplicon sequencing. All dormitory rooms were naturally ventilated. Information on cleaning frequency was collected but excluded from the final analysis due to the subjective nature of self-reported data.

Health data collection

A self-administered questionnaire was distributed to the 357 student occupants residing in the sampled dormitory rooms (97.3% participation rate). The questionnaire collected demographic information (gender and smoking habits) and assessed health outcomes using questions conceptually adapted from standard methodologies and translated into Chinese (see Appendix for the English and Chinese versions of the questionnaire). Asthma symptoms were assessed using the question: "In the last 12 months, have you had wheezing or whistling in the chest when you DID NOT have a cold or the flu?" (adapted from the International Study of Asthma and Allergies in Childhood (ISAAC)) [16]. Respiratory infections were assessed by asking: "Have you had a respiratory infection within the last 3 months?" (adapted from the European Community Respiratory Health Study (ECRHS)) [17]. The study protocol was reviewed and approved by the Medical Ethics Committee of Fudan University, Shanghai, China (approval no. 2017-11-0644). All participants provided written informed consent.

DNA extraction and 16S rRNA gene sequencing

Total genomic DNA was extracted from the dust supernatants using the E.Z.N.A. Soil DNA Kit (Omega Bio-Tek, Inc., GA, USA) according to the manufacturer’s protocol, which utilizes bead-beating and spin-filter techniques. A negative extraction control (reagents only) was processed alongside the samples and showed no visible amplification band via agarose gel electrophoresis. The V3-V4 hypervariable region of the 16S rRNA gene was amplified using primers 338F (5’-ACTCCTACGGGAGGCAGCA-3’) and 806R (5’-GGACTACHVGGGTWTCTAAT-3’). PCR was performed in a 25 μl reaction volume with Q5 High-Fidelity DNA Polymerase (New England Biolabs) for 25 cycles. PCR amplicons were purified using Agencourt AMPure XP beads (Beckman Coulter, USA) and quantified using the PicoGreen dsDNA Assay Kit (Invitrogen, USA). The pooled, barcoded library was sequenced on an Illumina MiSeq platform using a MiSeq Reagent Kit version 3 (Illumina, USA).

Bioinformatic processing and data analysis

Raw sequences were extracted according to the barcode information and filtered with the following criteria: minimum reads sequence length >150 bp, average Phred score >20, contained no ambiguous bases, and no mononucleotide repeats >8 bp. Chimeric reads were removed by USEARCH (version 5.2.236, Robert C. Edgar (Independent Researcher), Tiburon, California, USA), and paired-end reads were assembled by FLASH (version 1.2.7, Center for Computational Biology, Johns Hopkins University, Baltimore, Maryland, USA) [18], with a minimum of 10 bp overlapping between forward and reverse reads without mismatches. The assembled high-quality reads were clustered into operational taxonomic units (OTUs) at 97% sequence similarity threshold by UCLUST (version 5.2.236, Robert C. Edgar (Independent Researcher), Tiburon, California, USA) [19]. A representative sequence from each OTU was selected to blast against the Silva ribosomal RNA database (release 115, Max Planck Institute for Marine Microbiology, Bremen, Germany) to acquire the taxonomic information by using the best hit. The following analyses were conducted in the Quantitative Insights Into Microbial Ecology (QIIME, version 1.8.0, Caporaso Lab, Northern Arizona University, Flagstaff, Arizona, USA / Knight Lab, University of Colorado, Boulder, Colorado, USA) platform [20] and R (version 3.4, R Core Team 2018). An OTU table was built to store the abundance for each taxonomic unit. The operational taxonomic unit threshold (c value) was set to 0.01% in QIIME, with other parameters following a previous suggestion.

For the alpha diversity analyses (observed ASVs and Shannon index), the samples were rarefied to an even depth of 11,000 reads per sample to normalize for sequencing effort. Differences in alpha diversity between sample types were assessed using the Wilcoxon rank-sum test. Beta diversity was calculated on the unrarefied data using the weighted UniFrac distance metric [21] and visualized with principal coordinate analysis (PCoA). The significance of compositional differences between sampling strategies and other environmental factors was tested using permutational multivariate analysis of variance (PERMANOVA) via the adonis function in the vegan package, with 999 permutations.

The functional potential of the bacterial communities was predicted from the ASV data using PICRUSt2 (version 2.4.1, Langille Lab, Dalhousie University, Halifax, Nova Scotia, Canada) [22]. LEfSe (linear discriminant analysis effect size, version 1.7, Huttenhower Lab, Harvard T.H. Chan School of Public Health, Boston, Massachusetts, USA) analysis was conducted to identify the differentially abundant taxa between the sampling strategies [23].

Statistical analysis for health associations

To assess the relationship between bacterial genera and occupant health, a hierarchical logistic regression model was used, with dormitory building included as a random effect to account for clustering. The models were adjusted for gender and smoking. Only genera with a mean relative abundance > 0.1% and present in at least 20 dormitory rooms were included in the analysis. For these exploratory analyses, associations with a p-value < 0.01 and a false discovery rate (FDR) corrected p-value < 0.2 were considered statistically significant. All health-related statistical modeling was performed in Stata Statistical Software (release 15.0, StataCorp LLC, College Station, TX).

## Results

Sequencing overview and quality control

We collected paired settled air dust (n = 86, 98.9%) and vacuumed surface dust (n = 83, 95.4%) samples from 87 university dormitory rooms. After quality filtering, merging, and chimera removal, the high-throughput sequencing of the 16S rRNA gene V3-V4 region yielded a total of 6,834,127 high-quality reads. The number of reads per sample ranged from 27,377 to 53,783 (interquartile range: 32,710-41,377). There was no significant difference in sequencing depth between the two sample types (Wilcoxon test, p > 0.05), ensuring a robust basis for comparison. Following clustering at 97% sequence similarity, a total of 3,616 unique operational taxonomic units (OTUs) were identified across all samples, mapping to 530 distinct genera.

Sampling method drives profound differences in bacterial diversity and composition

**Figure 1:**
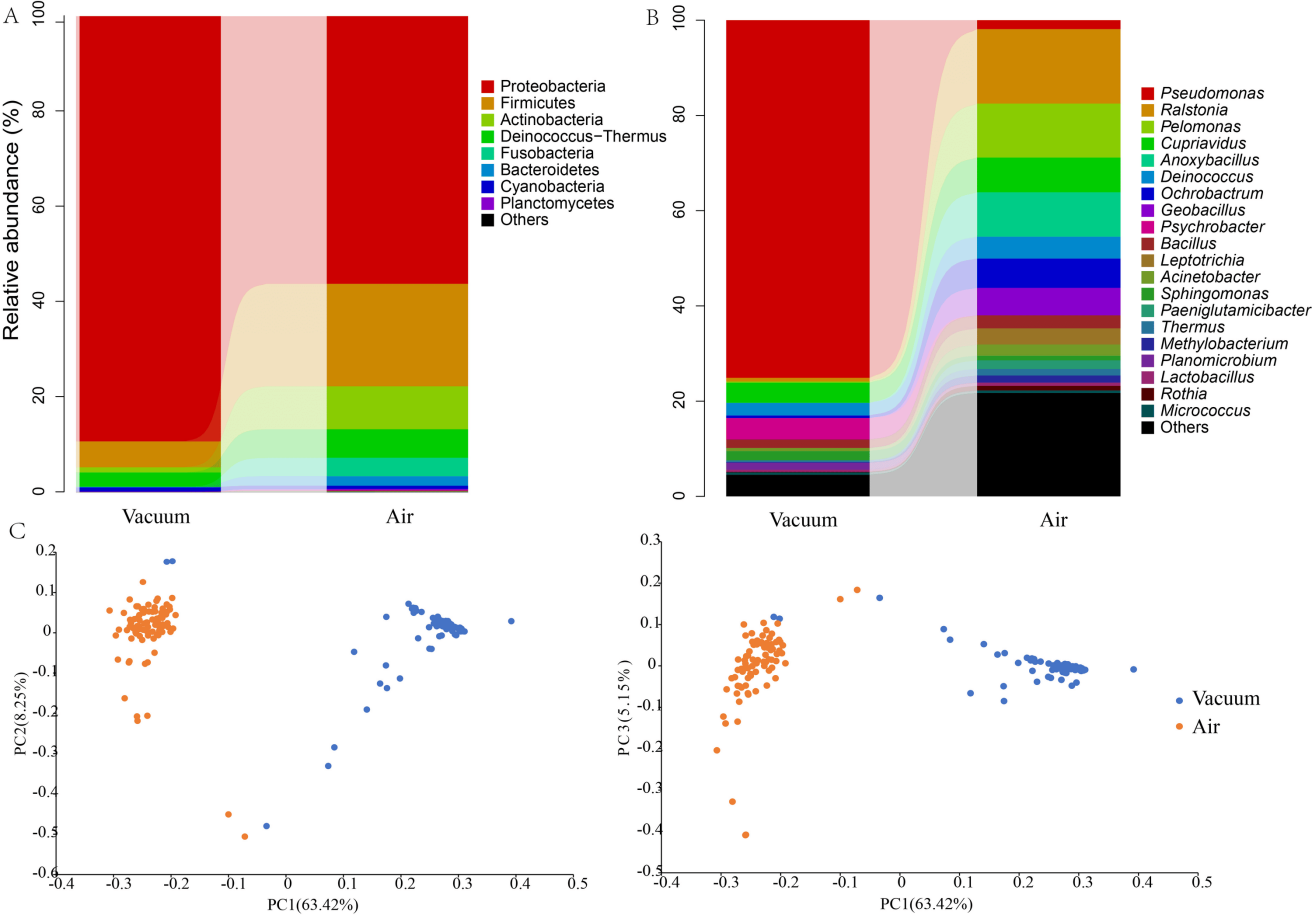
Distinct bacterial community composition in settled air dust and vacuumed surface dust. (A) Mean relative abundance of the top 10 bacterial phyla, comparing the two sampling methods. (B) Mean relative abundance of the top 15 most abundant bacterial genera, highlighting the dominance of *Pseudomonas* in vacuumed dust and the diverse community in air dust. (C) Principal coordinate analysis (PCoA) plot based on weighted UniFrac distances, illustrating the clear and significant separation of bacterial communities by sample type (PERMANOVA, R² = 0.65, p < 0.001). Each point represents an individual sample, colored by its source. The percentage of variation explained by each principal coordinate is shown in the axis labels.

The primary factor structuring the bacterial communities was the sampling method. Beta diversity analysis using weighted UniFrac distances revealed a stark and highly significant separation between air dust and vacuumed dust samples (Figure 1). This visual separation was statistically confirmed, with the sampling method explaining 65% of the total variance in bacterial composition (PERMANOVA, R² = 0.65, p < 0.001). Other environmental factors, including building age, location, and occupant sex, were not significantly associated with the observed microbiome variation (p > 0.05).

This compositional divergence was mirrored in the alpha diversity. Air dust samples were significantly richer and more diverse than vacuumed dust samples. The mean number of observed OTUs in air dust was 906.2 (± 179.6), more than double that of vacuumed dust at 345.1 (± 82.3) (Wilcoxon rank-sum test, p < 0.001).

Taxonomic profiles are distinct for each sampling method

The taxonomic compositions of the two sample types were fundamentally different at all levels (Figure 1A, 1B). Vacuumed dust samples were overwhelmingly dominated by the phylum Proteobacteria (mean relative abundance 89.3%). At the genus level, this was driven almost entirely by *Pseudomonas*, which alone accounted for 75.1% of the bacterial reads. Other less abundant genera included *Psychrobacter* (4.5%) and *Cupriavidus* (4.2%).

In stark contrast, air dust samples displayed a more even community structure. While still the most abundant phylum, Proteobacteria constituted a smaller portion of the community (56.2%), followed by Firmicutes (21.5%) and Actinobacteria (9.0%). The most abundant genera in air dust were *Ralstonia* (15.6%), *Pelomonas* (11.3%), *Anoxybacillus* (9.3%), and *Cupriavidus* (7.3%). Notably, the dominant genus from vacuumed dust, *Pseudomonas*, was only a minor component of the air dust community, accounting for just 1.9% of reads. Using LEfSe to formally test for differential abundance, we confirmed that *Pseudomonas* and *Psychrobacter* were significantly enriched in vacuumed dust, while all top 10 genera from air dust, including *Ralstonia* and *Pelomonas*, were significantly enriched in that sample type (p < 0.05).

Predicted functional profiles differ between sample types

**Figure 2:**
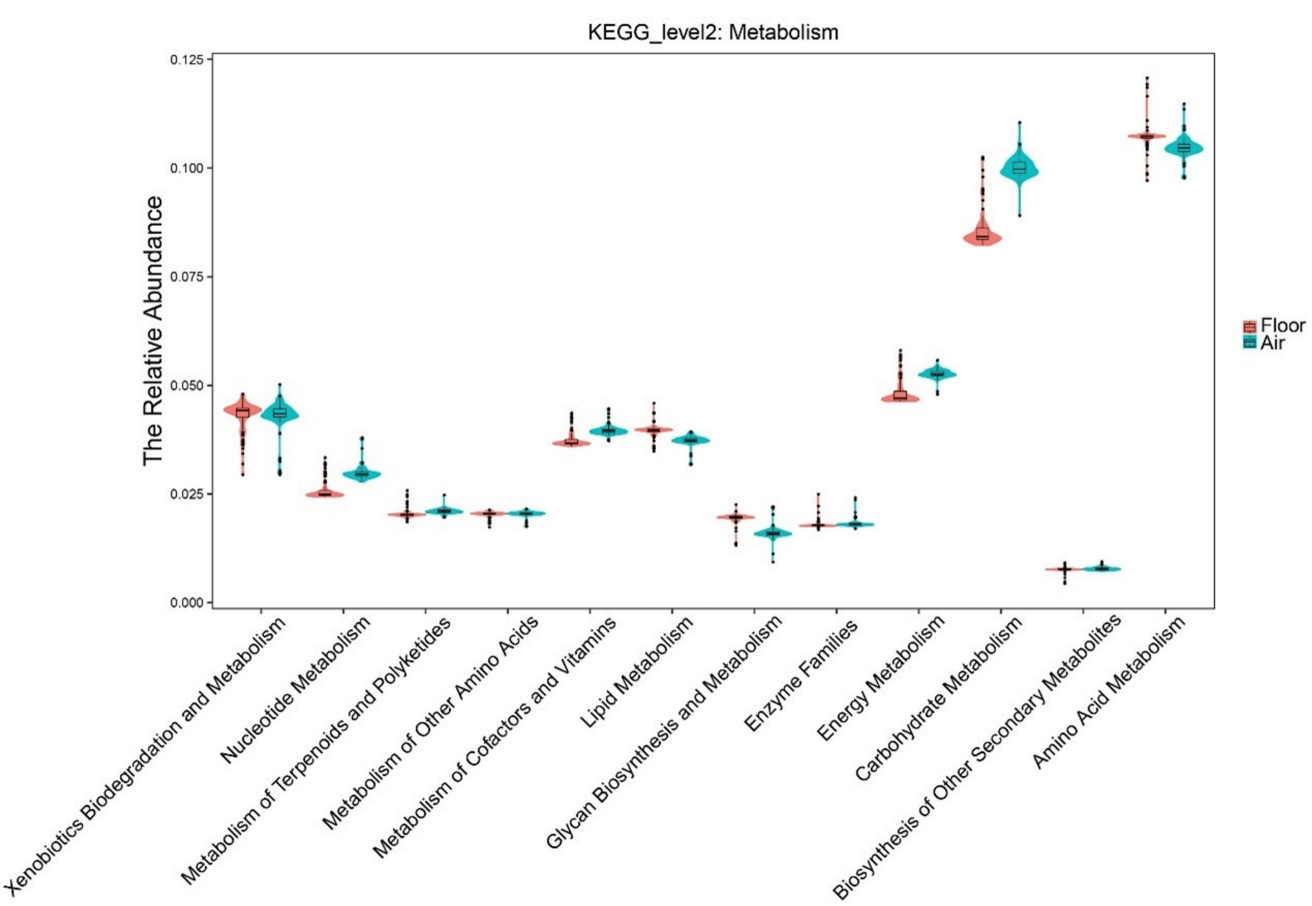
Predicted metabolic functional profiles of bacterial communities. The relative abundance of major KEGG level 2 metabolic pathways was predicted using PICRUSt2. The plot compares pathway abundances between settled air dust (blue) and vacuumed surface dust (red) samples. Violin plots show the density distribution of the data, with the internal boxplot representing the median and interquartile range (IQR). Significant differences (t-test, p < 0.05) were observed for several pathways, including the enrichment of carbohydrate metabolism and energy metabolism in air dust and the enrichment of lipid metabolism in vacuumed dust.

**Figure 3:**
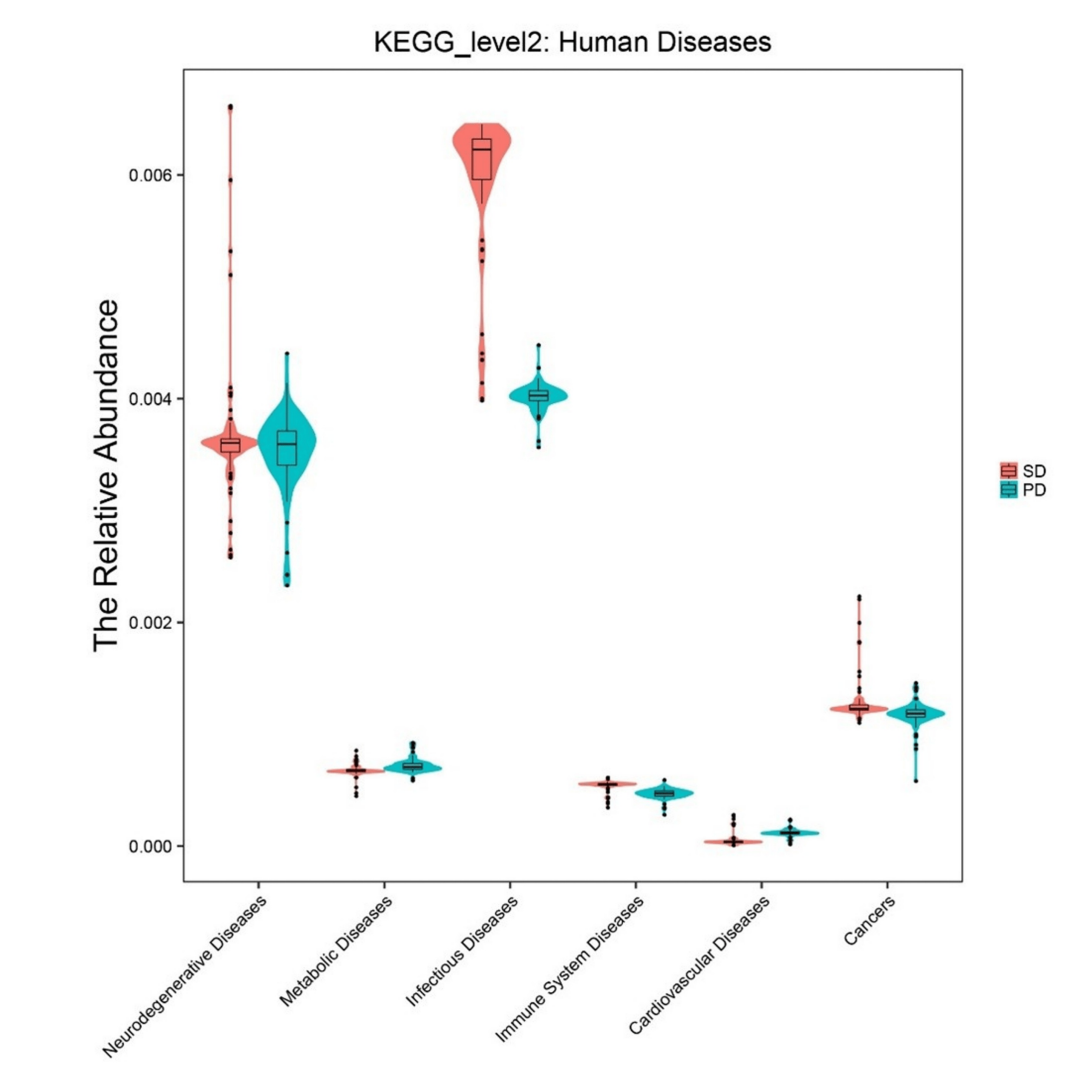
Predicted disease-related pathway profiles of bacterial communities. The relative abundance of KEGG level 2 human disease-related pathways was predicted using PICRUSt2. The plot compares predicted pathway abundances between settled air dust (blue) and vacuumed surface dust (red) samples. Violin plots show the data distribution, median, and IQR. Significant differences (t-test, p < 0.01) were observed for several pathways, with genes related to infectious diseases enriched in vacuumed dust and genes related to immune system diseases enriched in air dust.

To investigate if the taxonomic differences translated to functional differences, we used PICRUSt to predict the functional potential of the bacterial communities. The analysis revealed significant differences in the abundance of multiple KEGG metabolic pathways (Figure 2). For example, pathways related to *Carbohydrate Metabolism* and *Energy Metabolism* were predicted to be significantly more abundant in the diverse air dust communities (t-test, p < 0.01). Conversely, pathways for *Metabolism of Other Amino Acids* and *Lipid Metabolism* were significantly enriched in the *Pseudomonas*-dominated vacuumed dust samples (t-test, p < 0.01). Analysis of predicted disease-related pathways also showed significant differences, with genes related to* Infectious Diseases and Cardiovascular Diseases* being more abundant in vacuumed dust, while *Immune System Diseases *pathways were enriched in air dust (Figure 3, all p < 0.01).

Sampling method determines associations with occupant health

To illustrate the epidemiological consequences of sampling choice, we performed exploratory analyses linking bacterial genera to self-reported asthma and respiratory infections. The results were entirely dependent on the sampling method.

For asthma, we identified several significant associations within the air dust samples. The genus *Collinsella* was associated with a more than two-fold increase in the odds of asthma (OR = 2.17; 95% CI (1.3, 3.62); p = 0.003), while *Paracoccus* was strongly associated with decreased odds (OR = 0.062; 95% CI (0.008, 0.45); p = 0.006) (Table 1). In vacuumed surface dust, three genera from the phylum Firmicutes (*Peptoclostridium*, *Subdoligranulum*, and *Megamonas*) were associated with asthma. However, the odds ratios (ORs) for these taxa were low (1.04-1.09) compared to the stronger associations observed in air dust (Table 1).

**Table 1:** Associations between bacterial genera and asthma prevalence. Results are shown for bacterial genera identified in settled air dust samples. No significant associations were found for any genera in vacuumed surface dust. Associations were determined using a hierarchical logistic regression model, adjusted for gender and smoking, with dormitory building included as a random effect. Only genera with a mean relative abundance > 0.1% in at least 20 rooms and a false discovery rate (FDR) < 0.2 are presented. Abbreviations: OR, odds ratio; 95% CI, 95% confidence interval; FDR, false discovery rate

Sample type	Phylum	Class	Genus	OR (95% CI)	p-value	False discovery rate
Settled air dust	Actinobacteria	Coriobacteriia	Collinsella	2.17 (1.3, 3.62)	0.003	0.099
	Proteobacteria	Alphaproteobacteria	Paracoccus	0.062 (0.008, 0.45)	0.006	0.13
		Gammaproteobacteria	Enhydrobacter	0.004 (0, 0.13)	0.002	0.099
Vacuum surface dust	Firmicutes	Clostridia	Peptoclostridium	1.07 (1.02, 1.12)	0.004	0.03
			Subdoligranulum	1.09 (1.03, 1.15)	0.004	0.03
		Negativicutes	Megamonas	1.04 (1.01, 1.06)	0.004	0.03

A similar pattern emerged for respiratory infections. In air dust, four bacterial genera were significantly associated with an increased risk of infection, including *Slackia* (OR = 5.94; 95% CI (1.73, 20.33); p = 0.005) and *Ruminiclostridium* (OR = 1.39; 95% CI (1.1, 1.76); p = 0.006) (Table 2). Once again, no bacterial genera from the vacuumed dust samples showed any significant association with respiratory infections.

**Table 2:** Associations between bacterial genera and respiratory infection prevalence. Results are shown for bacterial genera identified in settled air dust samples. No significant associations were found for any genera in vacuumed surface dust. Associations were determined using a hierarchical logistic regression model, adjusted for gender and smoking, with dormitory building included as a random effect. Only genera with a mean relative abundance > 0.1% in at least 20 rooms and a false discovery rate (FDR) < 0.2 are presented. Abbreviations: OR, odds ratio; 95% CI, 95% confidence interval; FDR, false discovery rate

Sample type	Phylum	Class	Genus	OR (95%CI)	p-value	False discovery rate
Settled air dust	Actinobacteria	Coriobacteriia	Slackia	5.94 (1.73-20.33)	0.005	0.11
	Bacteroidetes	Cytophagia	Cytophaga	1.21 (1.05-1.38)	0.008	0.11
	Firmicutes	Clostridia	Ruminiclostridium	1.39 (1.1-1.76)	0.006	0.11
		Negativicutes	Phascolarctobacterium	1.43 (1.17-1.75)	0.001	0.07

## Discussion

This study provides a direct comparison of two common sampling methods for indoor bacterial communities and demonstrates that the choice of method is a critical determinant of scientific outcomes. We found that settled air dust and vacuumed surface dust from the same university dormitory rooms yielded profoundly different bacterial profiles, which in turn led to completely contradictory conclusions in exploratory epidemiological analyses. Our findings underscore that these methods are not interchangeable and must be selected based on a clear hypothesis regarding the relevant route of exposure. 

The most striking finding was the overwhelming dominance of *Pseudomonas* in vacuumed surface dust, a phenomenon that drove the significantly lower alpha diversity in these samples. *Pseudomonas* is a ubiquitous genus found in soil and water, and its prevalence in floor dust is likely due to being tracked indoors on shoes and introduced via tap water during daily mopping [24,25]. The vacuuming method is highly efficient at collecting larger, settled particles from surfaces, and *Pseudomonas* bacteria often attach to such particles [26,27]. This extreme dominance likely created a "dominant distortion" effect, a known artifact where the high biomass of a single taxon swamps the sequencing reads, artificially deflating diversity metrics and masking the presence of rarer, but potentially important, microbes [28]. This suggests that while vacuumed dust is an excellent integrator of total surface microbial load, its richness estimates can be misleading in environments with a single, overwhelmingly dominant taxon.

By contrast, the settled air dust samples provided a much different and more diverse snapshot of the indoor environment. This profile likely represents a mixture of smaller, aerosolized particles from various sources, including soil, human occupants, and outdoor air infiltration. These particles are small enough to remain suspended in the air and settle on elevated surfaces, making them a more plausible proxy for inhalable exposure [13]. The distinct bacterial community found in air dust, rich in genera like *Ralstonia* and *Pelomonas*, highlights that the fraction of the microbiome that is airborne is compositionally distinct from the total reservoir found on surfaces.

The epidemiological consequences of these methodological differences were profound. We identified several bacterial genera in air dust significantly associated with asthma and respiratory infections, including *Collinsella* and *Slackia* as risk factors and *Paracoccus* as a protective factor. While we identified three asthma-associated taxa in vacuumed dust, their effect sizes were small (OR < 1.1) compared to the stronger associations found in air dust (OR > 2.0). Furthermore, no vacuumed dust taxa were associated with respiratory infections. This does not imply that surface dust is irrelevant to health, but rather that for respiratory conditions mediated by inhalation, the airborne fraction of the microbiome may be the more biologically relevant exposure to measure. Many influential studies on childhood asthma have successfully used vacuumed floor or mattress dust [5,6,29], but our results suggest that concurrent analysis of air dust could provide a complementary and perhaps more direct picture of the specific microbes linked to respiratory outcomes.

This study has several important limitations. First, its cross-sectional design can only identify associations and cannot establish causality. We cannot determine whether microbial exposures lead to health conditions or if the conditions themselves (or related behaviors) alter the microbiome. Second, our health data were based on self-reported questionnaires, which can be subject to recall bias, rather than on clinical diagnoses. Third, analyzing low-biomass samples like settled air dust presents inherent challenges, as they are more susceptible to the influence of background DNA from laboratory reagents and the environment. Therefore, findings related to taxa in these samples should be interpreted with this context in mind, and future studies would be strengthened by including fully sequenced negative controls to better characterize potential background signals. Fourth, the prediction of microbial function using PICRUSt2 is an inference based on a marker gene and not a direct measurement of community function, which would require shotgun metagenomics. Fifth, our study was conducted in a specific setting (university dormitories in Northern China), so the findings may not be generalizable to other building types, climates, or geographical regions. Future multi-center studies are needed to validate these results in diverse environments. Finally, while we adjusted for key demographics, the association with respiratory infections may be influenced by unmeasured confounders such as outdoor air pollution exposure, viral coinfections, or seasonal variations that were not fully controlled for in this study design.

## Conclusions

Our study demonstrates that sampling strategy is not a trivial detail but a critical variable that can fundamentally alter the conclusions of indoor microbiome research. Settled air dust, likely representing inhalable exposure, and vacuumed surface dust, representing a long-term reservoir, are not interchangeable. They capture distinct bacterial communities, and as we have shown, this can lead to completely different outcomes when investigating links to respiratory conditions like asthma. A study relying solely on vacuumed dust might incorrectly conclude no association exists, while one using air dust might find several. To ensure valid health risk assessments, we recommend that future epidemiological studies and indoor air quality guidelines consider a multi-method sampling approach to capture a comprehensive profile of microbial exposure. Aligning sampling strategies with specific exposure hypotheses, particularly distinguishing between inhalable air and surface reservoirs, is essential for accurate data interpretation and effective policy implementation.

## Appendices

Questionnaire items used in the study

English Version

The following questions were translated into Chinese and distributed to participants. Questions regarding asthma were conceptually adapted from the International Study of Asthma and Allergies in Childhood (ISAAC) [16], and the question regarding respiratory infection was adapted from the European Community Respiratory Health Study (ECRHS) [17].

Category: Question / Item

Demographics: Gender (Male / Female)

Smoking habit: Do you smoke? (Yes / No)

Asthma: In the last 12 months, have you had wheezing or whistling in the chest when you DID NOT have a cold or the flu?

Rhinitis: In the last 12 months, have you had a problem with sneezing, a runny nose, or a blocked nose when you did not have a cold or the flu?

Respiratory infection: Have you had a respiratory infection within the last three months?

Chinese Version

大学生健康与宿舍空气品质的研究

本问卷调查结果统一由学校进行保密处理，数据信息仅做科研使用，根据《中华人民共和 国保密法》，对您填写的信息我们会严格保密！请回答您的真实情况!

姓名___________年级_________ 班级_________年龄 _________性别 _____ 你的身高________厘米； 体重________公斤

（一） 有关呼吸有杂声或哮鸣音(尖哨声)的问题：

你是否发生过呼吸时胸部发出杂声或哮鸣音(尖哨声)？ 是 ( ) 否( )

在最近十二个月里, 你是否出现过呼吸有杂声或哮鸣音(尖哨声)？是 ( ) 否 ( )

你是否患过哮喘? 是 ( ) 否 ( )

在最近十二个月里，你是否发过哮喘? 有 ( ) 否 ( )

在最近三个月里，你是否有过上呼吸道感染? 是 ( ) _________次 否 ( )

（二） 有关鼻症状的问题：

你是否在未感冒的情况下有打喷嚏，流鼻涕及鼻塞的症状? 有 ( ) 否 ( )

在最近十二个月里, 你是否在未感冒的情况下有打喷嚏，流鼻涕及鼻塞的症状? 有 ( ) 否（ )
